# Supplementary material for: A Poisson-multinomial model for quantifying gene therapy vector integrity using quadplex digital PCR
Source: PLoS One. 2026 Nov 12;21(11):e0362653. doi: 10.1371/journal.pone.0362653 (PMC13759107; doi:10.1371/journal.pone.0362653)
Supplement: Supplementary file 1 [file pone.0362653.s012.pdf]

S1 Table. Sample preparation of CMV deletion variant mixtures. Samples prepared as mixtures of pLV-CMV-EGFP MfeI digests (100% intact) and ΔCMV deletion variant linearized with MfeI and their expected target amplification probabilities.

Sample Number	pLV-CMV-EGFP MfeI digest (%)	ΔCMV (%)	Expected p1111
Sample 1	100	0	100
Sample 2	75	25	75
Sample 3	50	50	50
Sample 4	25	75	25
Sample 5	0	100	0
