# Supplementary material for: A Poisson-multinomial model for quantifying gene therapy vector integrity using quadplex digital PCR
Source: PLoS One. 2026 Nov 12;21(11):e0362653. doi: 10.1371/journal.pone.0362653 (PMC13759107; doi:10.1371/journal.pone.0362653)
Supplement: Supplementary file 2 [file pone.0362653.s013.pdf]

S2 Table. Sample preparation of WPRE deletion variant mixtures. Samples prepared as mixtures of pLV-CMV-EGFP MfeI digests (100% intact) and ΔWPRE deletion variant linearized with MfeI and their expected target amplification probabilities.

Sample Number	pLV-CMV-EGFP MfeI digest (%)	ΔWPRE (%)	Expected p1111
Sample 6	100	0	100
Sample 7	75	25	75
Sample 8	50	50	50
Sample 9	25	75	25
Sample 10	0	100	0
