# Supplementary material for: A Poisson-multinomial model for quantifying gene therapy vector integrity using quadplex digital PCR
Source: PLoS One. 2026 Nov 12;21(11):e0362653. doi: 10.1371/journal.pone.0362653 (PMC13759107; doi:10.1371/journal.pone.0362653)
Supplement: Supplementary file 3 [file pone.0362653.s015.pdf]

S3 Table. Summary results of model estimates for deletion variant mixtures. Average model estimates of percent intact and fragmented genomes corresponding to S12 Fig, n=2.

Sample	Modeled %intact and fragmented genomes		
	p1111	p1011	p1110
S1	90.9%	0.4%	0.4%
S2	65.0%	22.0%	0.2%
S3	41.9%	42.4%	0.1%
S4	20.4%	60.8%	0.0%
S5	-2.3%	81.3%	0.0%
S6	91.7%	0.3%	0.2%
S7	70.9%	0.3%	20.5%
S8	49.3%	0.2%	40.3%
S9	24.6%	0.0%	65.0%
S10	-2.1%	0.0%	90.7%
