# Supplementary material for: A Poisson-multinomial model for quantifying gene therapy vector integrity using quadplex digital PCR
Source: PLoS One. 2026 Nov 12;21(11):e0362653. doi: 10.1371/journal.pone.0362653 (PMC13759107; doi:10.1371/journal.pone.0362653)
Supplement: Supplementary file 4 [file pone.0362653.s024.pdf]

S4 Table. Comparison of long-read NGS and quadplex model integrity estimates. Extracted ssAAV9-BIIB1 genomes were annealed and purified to generate duplex templates suitable for sequencing. The same purified duplex samples were analyzed by long-read NGS and by quadplex droplet dPCR followed by quadplex model analysis. Resulting estimates of percentage of intact genomes are shown for method comparison.

Material	Condition	intact rate NGS (%) n=1	intact rate ddPCR quadplex model of NGS samples (% n=1)
AAV9-BIIB1	Control	87	68
	5 min 95°C	78	59
	15 min 95°C	38	22
