# Supplementary material for: A Poisson-multinomial model for quantifying gene therapy vector integrity using quadplex digital PCR
Source: PLoS One. 2026 Nov 12;21(11):e0362653. doi: 10.1371/journal.pone.0362653 (PMC13759107; doi:10.1371/journal.pone.0362653)

S1 Raw images. Raw images of gels featured in S7, S8 and S21 Figs.

S7 Fig. (Plasmid sample preparation) was generated from this raw image. pLV-CMV-EGFP digests were run on a 1% agarose gel and visualized with SYBR Safe (Invitrogen) on an Odessey M imaging system (Licor). From left to right: Lane 1: NEB 1KB ladder (catalog N0552S); Lane 2: MfeI; Lane 3: MfeI and PacI; Lane 4: MfeI and EagI; Lane 5: MfeI, PacI, and EagI. Digested bands were of expected sizes (Table 2). Lanes 6-10 comprise duplicates for all Lanes 1-5. As they are redundant, these lanes were cropped from S7 Fig and have been marked with and X.

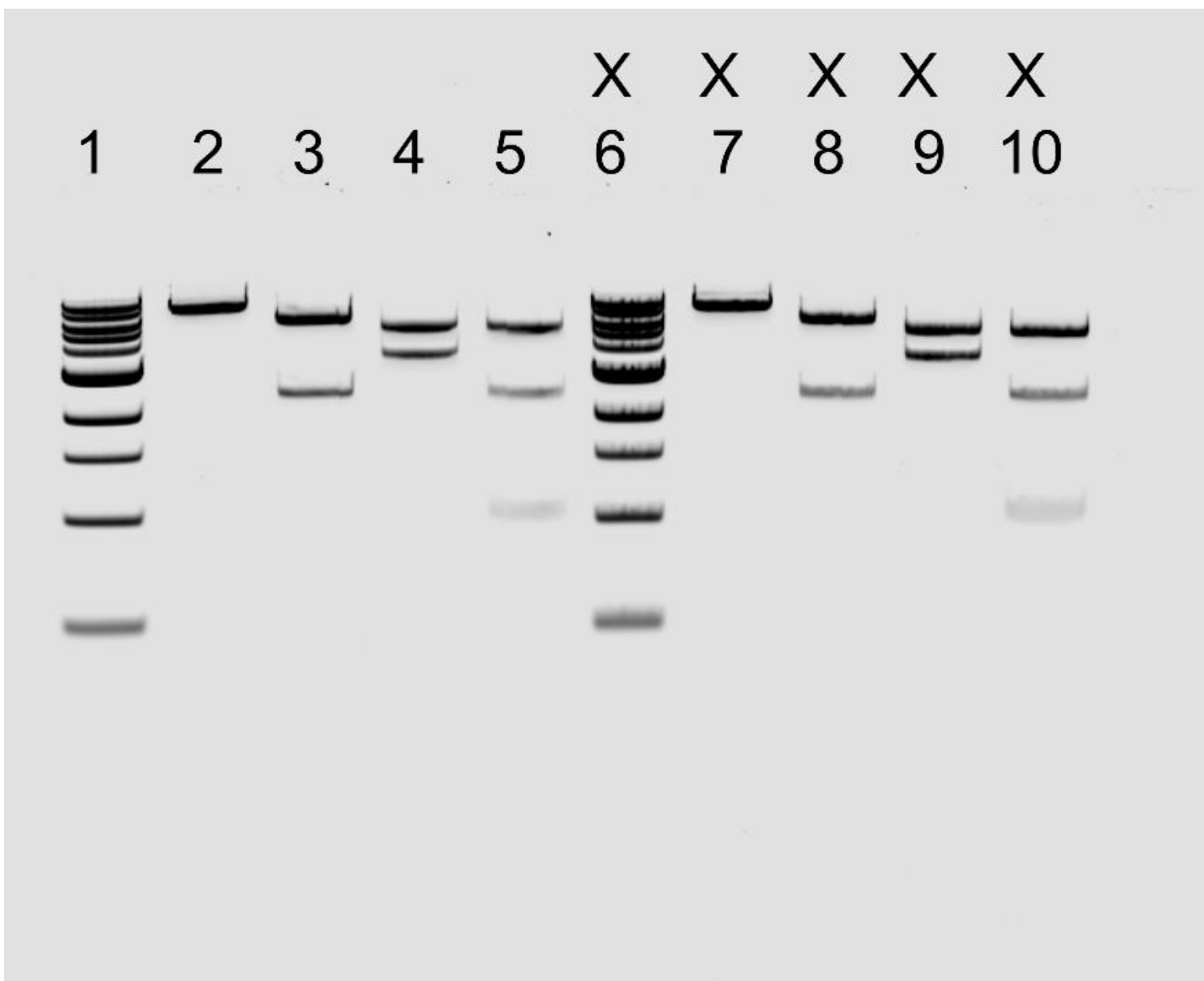

S8 Fig. (Plasmid sample preparation (continued) was generated from this raw image. pLV-CMV-EGFP digests were run on a 1% agarose gel and visualized with SYBR Safe (Invitrogen) on an Odessey M imaging system (Licor). From left to right: Lane 1: NEB 1KB ladder (catalog N0552S); Lane 2: MfeI and SnaBI; Lane 3: MfeI, SnaBI and PacI; Lane 4: MfeI, SnaBI and EagI; Lane 5: MfeI, SnaBI, PacI, and EagI (each corresponds to Fig 3B, E, F, H respectively). Digested bands were of expected sizes (Table 2). Lanes 6-9 are not relevant to any figures in the manuscript. These lanes have been marked with and X, and were cropped from S8 Fig.

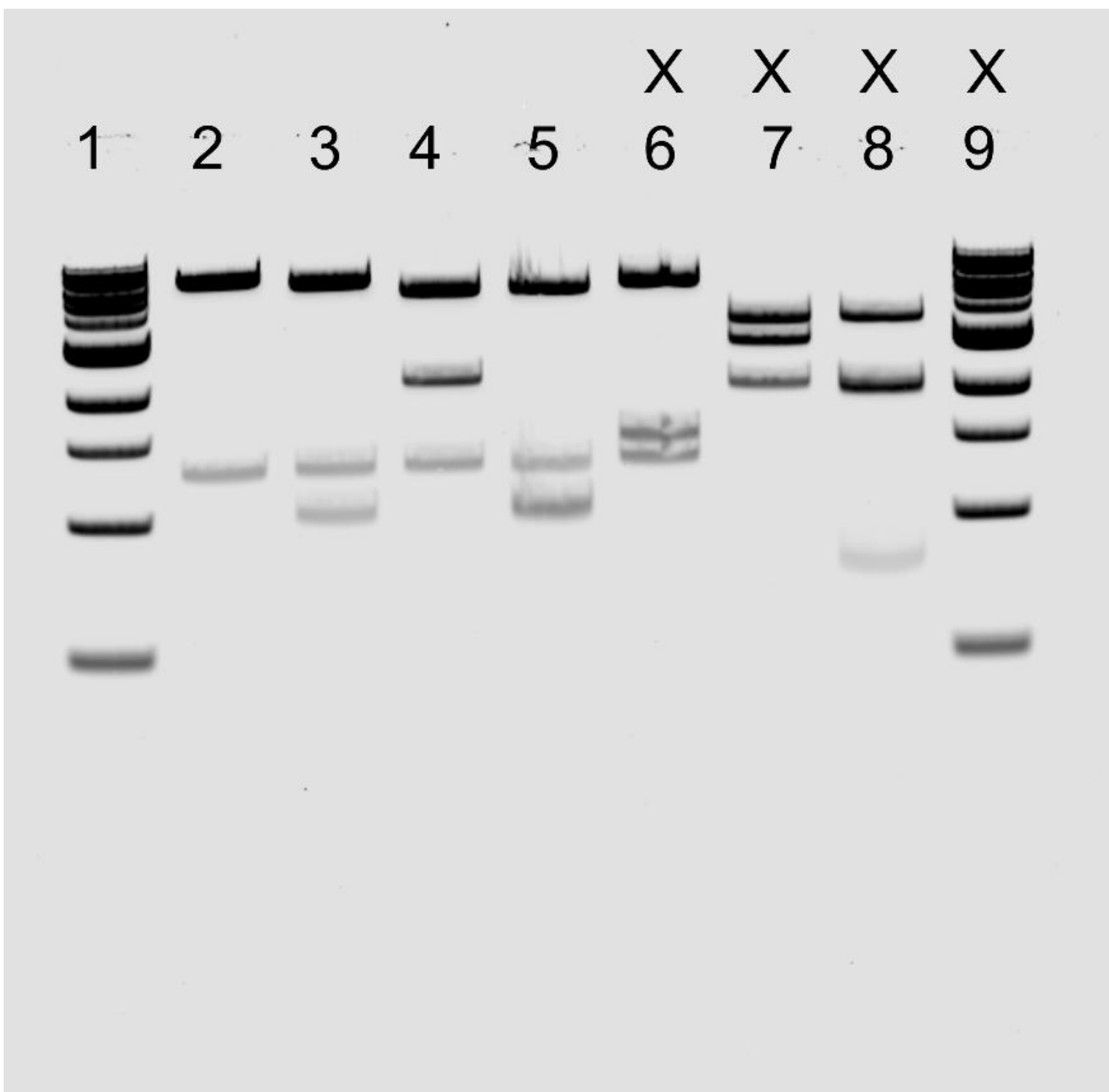

S21 Fig. (Long-read NGS samples visualized with gel electrophoresis) was generated from this raw image. Extracted and annealed dsDNA templates for control and stressed AAV9-BIIB1 were run on a 1% agarose gel and visualized with SYBR Safe (Invitrogen) on an Odessey M imaging system (Licor). From left to right: Lane 1: NEB 1KB ladder (catalog N0552S); Lane 2 AAV9-BIIB1 Control; Lane 3: AAV9-BIIB1 5 minutes 95°C; Lane 4: AAV9-BIIB1 15 minutes 95°C.

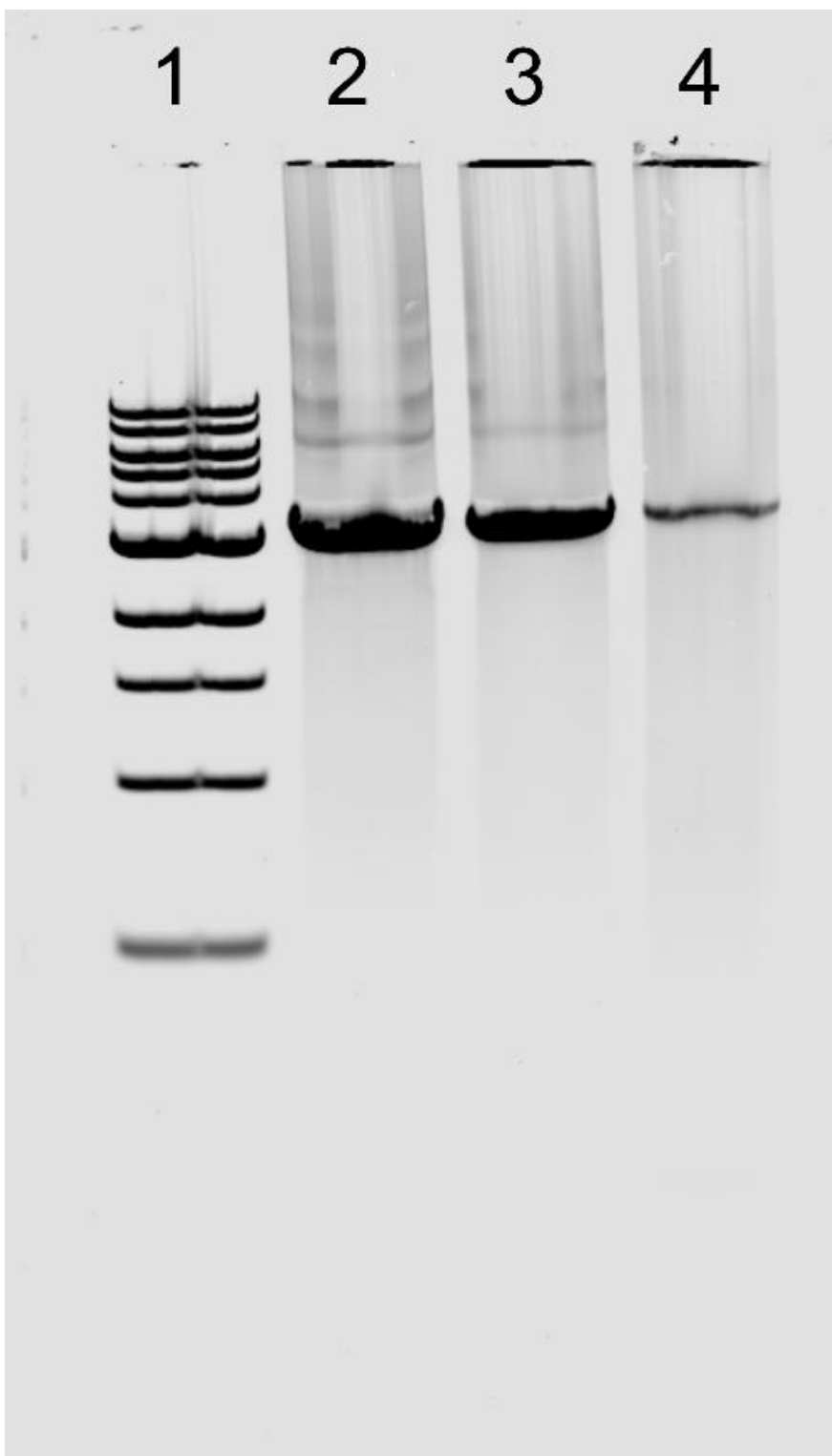

Supplement: Supplementary file 5 [file pone.0362653.s026.pdf]
