# Supplementary material for: A Poisson-multinomial model for quantifying gene therapy vector integrity using quadplex digital PCR
Source: PLoS One. 2026 Nov 12;21(11):e0362653. doi: 10.1371/journal.pone.0362653 (PMC13759107; doi:10.1371/journal.pone.0362653)
Supplement: Peer review history

# Author comment: Author response to Decision Letter 0

**DOI:** 10.1371/journal.pone.0362653.r001

**Transfer Alert**

This paper was transferred from another journal. As a result, its full editorial history (including decision letters, peer reviews and author responses) may not be present.

8 Jun 2026

# Peer review: Decision Letter 0

**DOI:** 10.1371/journal.pone.0362653.r002

**Authors:** Chen Ling

20 Aug 2026

Dear Dr. Tereshko,

Thank you for submitting your manuscript to PLOS One. After careful consideration, we feel that it has merit but does not fully meet PLOS One’s publication criteria as it currently stands. Therefore, we invite you to submit a revised version of the manuscript that addresses the points raised during the review process.

Please submit your revised manuscript by Oct 19 2026 11:59PM. If you will need more time than this to complete your revisions, please reply to this message or contact the journal office at plosone@plos.org. When you're ready to submit your revision, log on to https://www.editorialmanager.com/pone/ and select the 'Submissions Needing Revision' folder to locate your manuscript file.

- A letter that responds to each point raised by the academic editor and reviewer(s). You should upload this letter as a separate file labeled 'Response to Reviewers'.
- A marked-up copy of your manuscript that highlights changes made to the original version. You should upload this as a separate file labeled 'Revised Manuscript with Track Changes'.
- An unmarked version of your revised paper without tracked changes. You should upload this as a separate file labeled 'Manuscript'.

If you would like to make changes to your financial disclosure, please include your updated statement in your cover letter. Guidelines for resubmitting your figure files are available below the reviewer comments at the end of this letter.

If applicable, we recommend that you deposit your laboratory protocols in protocols.io to enhance the reproducibility of your results. Protocols.io assigns your protocol its own identifier (DOI) so that it can be cited independently in the future. For instructions see: https://journals.plos.org/plosone/s/submission-guidelines#loc-laboratory-protocols. Additionally, PLOS One offers an option for publishing peer-reviewed Lab Protocol articles, which describe protocols hosted on protocols.io. Read more information on sharing protocols at https://plos.org/protocols?utm_medium=editorial-email&utm_source=authorletters&utm_campaign=protocols.

As the corresponding author, your ORCID iD is verified in the submission system and will appear in the published article. PLOS supports the use of ORCID, and we encourage all coauthors to register for an ORCID iD and use it as well. Please encourage your coauthors to verify their ORCID iD within the submission system before final acceptance, as unverified ORCID iDs will not appear in the published article. *Only*  the individual author can complete the verification step; PLOS staff *cannot*  verify ORCID iDs on behalf of authors.

We look forward to receiving your revised manuscript.

Kind regards,

Chen Ling, Ph.D.

Academic Editor

PLOS One

Journal Requirements:

When submitting your revision, we need you to address these additional requirements.

1. Please ensure that your manuscript meets PLOS ONE's style requirements, including those for file naming. The PLOS ONE style templates can be found at https://journals.plos.org/plosone/s/file?id=wjVg/PLOSOne_formatting_sample_main_body.pdf and https://journals.plos.org/plosone/s/file?id=ba62/PLOSOne_formatting_sample_title_authors_affiliations.pdf

2. In the Methods, please ensure you state the commercial source/company that supplied the primary cells used in this study.

3. Thank you for stating the following in the Competing Interests section:

[I have read the journal's policy and the authors of this manuscript have the following competing interests: Biogen employees receive salaries and hold stock options as part of standard compensation.].

We note that one or more of the authors are employed by commercial companies: Biogen and Cencora Global Consulting Services.

1. Please provide an amended Funding Statement declaring this commercial affiliation, as well as a statement regarding the Role of Funders in your study. If the funding organization did not play a role in the study design, data collection and analysis, decision to publish, or preparation of the manuscript and only provided financial support in the form of authors' salaries and/or research materials, please review your statements relating to the author contributions, and ensure you have specifically and accurately indicated the role(s) that these authors had in your study. You can update author roles in the Author Contributions section of the online submission form.

Please also include the following statement within your amended Funding Statement.

“The funder provided support in the form of salaries for authors [insert relevant initials], but did not have any additional role in the study design, data collection and analysis, decision to publish, or preparation of the manuscript. The specific roles of these authors are articulated in the ‘author contributions’ section.”

If your commercial affiliation did play a role in your study, please state and explain this role within your updated Funding Statement.

2. Please also provide an updated Competing Interests Statement declaring this commercial affiliation along with any other relevant declarations relating to employment, consultancy, patents, products in development, or marketed products, etc.

Within your Competing Interests Statement, please confirm that this commercial affiliation does not alter your adherence to all PLOS ONE policies on sharing data and materials by including the following statement: "This does not alter our adherence to PLOS ONE policies on sharing data and materials.” (as detailed online in our guide for authors http://journals.plos.org/plosone/s/competing-interests). If this adherence statement is not accurate and there are restrictions on sharing of data and/or materials, please state these. Please note that we cannot proceed with consideration of your article until this information has been declared.

Please include both an updated Funding Statement and Competing Interests Statement in your cover letter. We will change the online submission form on your behalf.

4. PLOS ONE now requires that authors provide the original uncropped and unadjusted images underlying all blot or gel results reported in a submission’s figures or Supporting Information files. This policy and the journal’s other requirements for blot/gel reporting and figure preparation are described in detail at https://journals.plos.org/plosone/s/figures#loc-blot-and-gel-reporting-requirements and https://journals.plos.org/plosone/s/figures#loc-preparing-figures-from-image-files. When you submit your revised manuscript, please ensure that your figures adhere fully to these guidelines and provide the original underlying images for all blot or gel data reported in your submission. See the following link for instructions on providing the original image data: https://journals.plos.org/plosone/s/figures#loc-original-images-for-blots-and-gels.

In your cover letter, please note whether your blot/gel image data are in Supporting Information or posted at a public data repository, provide the repository URL if relevant, and provide specific details as to which raw blot/gel images, if any, are not available. Email us at plosone@plos.org if you have any questions.

If the reviewer comments include a recommendation to cite specific previously published works, please review and evaluate these publications to determine whether they are relevant and should be cited. There is no requirement to cite these works unless the editor has indicated otherwise.

[Note: HTML markup is below. Please do not edit.]

Reviewers' comments:

Reviewer's Responses to Questions

**Comments to the Author**

1. Is the manuscript technically sound, and do the data support the conclusions?

Reviewer #1: Yes

Reviewer #2: Yes

**********

2. Has the statistical analysis been performed appropriately and rigorously?

Reviewer #1: Yes

Reviewer #2: Yes

**********

3. Have the authors made all data underlying the findings in their manuscript fully available?

The PLOS Data policy

Reviewer #1: Yes

Reviewer #2: Yes

**********

4. Is the manuscript presented in an intelligible fashion and written in standard English?

Reviewer #1: Yes

Reviewer #2: Yes

**********

Reviewer #1: The manuscript by Tereshko et al. describes a novel quadplex digital PCR (dPCR) approach coupled with a Poisson-multinomial model to quantify the sequence integrity and genetic linkage of gene therapy vectors. By expanding upon their previous duplex model, the authors demonstrated the method's accuracy using linearized and restriction-digested plasmid templates. Furthermore, they applied this framework to decapsidated recombinant AAV (rAAV) samples subjected to heat and UV degradation, showing a correlation between model-estimated genome integrity and functional protein expression. Overall, this tool offers a promising analytical framework for characterizing vector quality; however, additional experimental controls and orthogonal analytical validation are required to establish the robustness and generalizability of the proposed approach.

Major

1.The authors should analyze the same untreated and stressed rAAV samples using an orthogonal method, such as long-read sequencing or Southern blotting, and quantitatively compare the results with the full-length genome fraction estimated by the model.

2.The authors should generate or obtain rAAV preparations containing defined truncations or deletions, mix them at known ratios, and analyze them as blinded samples to evaluate the recovery, bias, and precision of the model's estimates for both intact and partial genomes.

3.The authors should perform a systematic study to compare the effects of stress applied before versus after capsid decapsidation, and evaluate how different lysis temperatures, durations, and SmaI treatment impact individual target recovery and the model's integrity estimates.

4.The authors should design at least one independent, non-overlapping primer/probe panel and analyze a subset of samples with both panels to quantitatively demonstrate that the estimated genome integrity values are robust to target placement.

5.The authors should independently quantify the plasmid stock concentrations using a validated singleplex dPCR and an orthogonal method like fluorometry. Using these well-characterized standards, a full assay performance evaluation should be conducted, including accuracy, repeatability, intermediate precision, linearity, and the reportable range.

Minor

1.The description of rcAAV in the second paragraph of the Introduction is somewhat brief. The authors should briefly describe its origin, its impact on product safety, and the principles of conventional detection methods, such as cell-based amplification assays, to provide readers with better context.

2.Including the actual fragment lengths in base pairs in Table 2 would help readers better understand the discussion on how fragment size affects model accuracy.

3.The authors should provide additional details on fluorescence compensation, cluster definition, and threshold setting, and include representative two-dimensional cluster plots to demonstrate assay specificity.

4.Language and terminology require editing throughout the manuscript. For example, "lipid nano particles" should be "lipid nanoparticles," and the use of units like "µL" and the capitalization of "Bio-Rad" should be made consistent.

5.The serotypes of AAV-BIIB1 and AAV-BIIB2 are not mentioned anywhere. Different serotypes have different capsid stabilities and genome release properties, which could affect decapsidation efficiency and consequently the model's estimates. It would be helpful to specify this.

6.In the first paragraph of the Discussion, the authors state "While triplex ('3D') linkage analysis has been recently published, the statistical framework was not fully described [32]." The authors should also consider PMID: 39820513. Additionally, in the second paragraph of the Introduction, where the authors discuss rAAV vector quality attributes, the citation of PMID: 38307819 is recommended providing more comprehensive literature support for the background on rAAV vector characterization and quality control.

7.The authors note in the Methods that a linear mixed-effects model with replicate as a random effect was evaluated, but only Pearson's r and R² are reported in the main text. Since the mixed model was run, it would be good to present those results as well.

Reviewer #2: The manuscript provided here builds on that 2023 framework by extending the statistical formulation from a duplex (2-target) model to a quadplex (4-target) model for quantifying gene therapy vector integrity. It was well written, technique sound, and timing for the community. However, I have the following major concerns:

1. The authors empirically establish a concentration range (39- 5000 copies/μL) and a negative droplet threshold (≥5000" " empty droplets) required for accurate model performance. While these operational recommendations are practical, using an absolute count of 5000 empty droplets lacks statistical rigor because the total number of accepted droplets (N) varies between wells/reactions (typically around 20,000). Therefore, an absolute cutoff of 5000 empty droplets does not hold consistently across varying total droplet counts. For the fitting plot in Figure 5, I strongly suggest re-parameterizing the x-axis using the proportion of positive droplets (k/N), the proportion of negative droplets ((N-k)/N), or the overall occupancy parameter λ.

2. The presentation of probability estimates in Figures 3 and 4 is somewhat confusing. For any given sample, should the sum of all modeled probabilities across all species equal 1 (100%)? If the model output sums to greater or less than 1, are these values normalized back to 1? Please clarify how probability constraints and normalization are mathematically operationalized within the algorithm, particularly when impossible or negative fragment probabilities arise.

3. In Section III (p. 12) and the Discussion (p. 18), the authors hypothesize that smaller template fragments physically fit into droplets in greater numbers than larger fragments, leading to model inaccuracy. However, the current experimental design does not directly isolate fragment size (e.g., comparing long fragments of several kilobases against short fragments of a few hundred base pairs at identical molar concentrations). From the raw data, the higher-fragmentation samples simply contain significantly lower empty droplet counts. This suggests that the observed bias may be driven by high target occupancy (λ) rather than physical exclusion/size effects. The authors should clarify this distinction and consider whether λ or the positive droplet ratio (k/N) is the primary governing parameter.

4. The Discussion should expand on how total target occupancy (λ) and negative droplet ratios govern model accuracy. The concentration dynamic range established for a single-target assay (39- 5000 copies/μL ) does not directly translate to multi-target quadplex assays, where the cumulative concentration of all fragments combined determines the total partition occupancy and false co-encapsulation rates.

My minor concerns:

1. In the raw dPCR data, several assays exhibit low-level false-positive droplets in non-template control (NTC) wells. Are these false positives attributable to primer-dimer formation, probe crosstalk, or sub-optimal thresholding settings?

2. The starting stock concentrations for plasmid preparations are relatively low (∼0.04 ng/μL). Were specific precautions taken to prevent non-specific surface adsorption during serial dilutions (e.g., using low-binding plasticware or adding carrier nucleic acids such as 10 ng/μL yeast tRNA)?

3. Although "ddPCR" is widely recognized, the manuscript references digital PCR technology more broadly. To maintain precise distinction between microfluidic/nanowell-based dPCR and droplet-based partitioning, consider using "droplet dPCR" or "dPCR" consistently throughout the text instead of mixing "ddPCR" and "dPCR".

4. Check typos in target parameter names throughout Section II and the manuscript text (e.g., pPuroR_AmpR vs. ppuroR_ampR).

**********

what does this mean?). If published, this will include your full peer review and any attached files.

If you choose “no”, your identity will remain anonymous but your review may still be made public.

**Do you want your identity to be public for this peer review?**  For information about this choice, including consent withdrawal, please see our Privacy Policy

Reviewer #1: **Yes:**  Chen Ling

Reviewer #2: No

**********

[NOTE: If reviewer comments were submitted as an attachment file, they will be attached to this email and accessible via the submission site. Please log into your account, locate the manuscript record, and check for the action link "View Attachments". If this link does not appear, there are no attachment files.]

To ensure your figures meet our technical requirements, please review our figure guidelines: https://journals.plos.org/plosone/s/figures

You may also use PLOS’s free figure tool, NAAS, to help you prepare publication quality figures: https://journals.plos.org/plosone/s/figures#loc-tools-for-figure-preparation.

NAAS will assess whether your figures meet our technical requirements by comparing each figure against our figure specifications.

# Author comment: Author response to Decision Letter 1

**DOI:** 10.1371/journal.pone.0362653.r003

19 Oct 2026

We thank the Editor and Reviewers for their careful evaluation of our manuscript and for their constructive comments. We have revised the manuscript to incorporate the Reviewer’s suggestions and believe these changes have improved the clarity and quality of the work. Please find our responses in-line to each comment (bolded) below.

Response to Reviewer #1

Major 1. The authors should analyze the same untreated and stressed rAAV samples using an orthogonal method, such as long-read sequencing or Southern blotting, and quantitatively compare the results with the full-length genome fraction estimated by the model.

We thank the reviewer for this suggestion. To provide an orthogonal assessment, we subjected AAV9-BIIB material to varying durations of 95°C heat stress and analyzed the samples with long-read PacBio sequencing. For this experiment, the purified samples sent for sequencing were also assayed by quadplex ddPCR and analyzed by the quadplex model, enabling direct comparison between methods.

Both long-read sequencing and the quadplex ddPCR model detected a similar stress-dependent decrease in estimated intact genomes. The absolute estimates differed modestly between methods, as each employs distinct operational definitions of “intact genomes”. The NGS analytical pipeline defines intact rate as the proportion of genome-containing reads covering ≥90% of the reference genome. In contrast, the quadplex ddPCR model estimates the fraction of molecules with linkage between all four targeted regions. Consequently, direct numerical agreement is not necessarily expected. Intact genome estimates obtained from the purified samples differed from the corresponding values reported in the main manuscript. This was expected as long-read sequencing requires conversion of single-stranded AAV genomes into duplex templates prior to library preparation. Annealing, purification and subsequent library construction steps can further alter the representation of fragmented genome species present relative to unpurified stressed rAAV samples.

We have added the quantitative comparison (S4 Table) and clarified “percentage of intact genome” definitions for each method in the revised manuscript. A representative agarose gel image (S21 Fig) has been included in the supplemental information which provides qualitative evaluation of the extracted and annealed duplex rAAV sent for sequencing. Consistent with the sequencing and quadplex ddPCR results, full-length genome band intensity decreased with increased stress duration providing qualitative evidence of progressive genome degradation.

Major 2. The authors should generate or obtain rAAV preparations containing defined truncations or deletions, mix them at known ratios, and analyze them as blinded samples to evaluate the recovery, bias, and precision of the model's estimates for both intact and partial genomes.

We appreciate the reviewer’s suggestion for further evaluation of defined deletion and truncated genome variants. To address this objective using materials with ≥90% intact templates, we elected to generate controlled plasmid deletion variants rather than rAAV variants which would contain variable proportions of intact and fragmented genomes.

Deletion variants derived from pLV-CMV-EGFP were generated with either the promoter or WPRE element deleted and mixed with the intact parental construct at known ratios. Despite <100% recovery of the respective expected p1011 and p1110 fragment types for ΔCMV and ΔWPRE controls, quadplex model estimates tracked with the expected input compositions, supporting the ability of the model to distinguish heterogeneous template populations under controlled conditions. The Results section has been edited, and these data have been added as S1 Table, S2 Table, S12 Fig, and S3 Table.

Major 3. The authors should perform a systematic study to compare the effects of stress applied before versus after capsid decapsidation, and evaluate how different lysis temperatures, durations, and SmaI treatment impact individual target recovery and the model's integrity estimates.

We thank the reviewer for this suggestion, however, we believe that while systematically evaluating stress timing and lysis conditions would provide useful information, it falls outside of the scope of the current work. The purpose of the heat and UV stressed rAAV experiments was to demonstrate application of the model to biologically relevant vectors with varying degrees of intact genomes. Additionally, SmaI treatment is known to improve the target recovery of elements bordering ITR regions (PMID: 36696399; PMID: 23828206) and was included in the dPCR reactions to facilitate amplification of regions near the ITRs. We have clarified this distinction in the revised manuscript.

Major 4. The authors should design at least one independent, non-overlapping primer/probe panel and analyze a subset of samples with both panels to quantitatively demonstrate that the estimated genome integrity values are robust to target placement.

To evaluate potential impact of target placement on model output, we analyzed plasmid intact control samples using an independent quadplex primer/probe panel targeting non-overlapping regions of the genome. Due to the limited size of rAAV genomes and the requirement to accommodate four targets while maintaining suitable assay performance, the alternative panel demonstrated reduced droplet separation and droplet cluster distortion consistent with inter-channel signal interference compared to the primary panel (S3-S6 Figs). Despite these limitations, similar model estimates of intact genomes for linear plasmid controls were obtained with the primary (mean = 96.4%) and alternative (mean = 93.2%) panels when evaluated at the same nominal dilution levels (625-1250 copies/μL) (S9 Fig). These data suggest that model estimates were not substantially altered by primer/probe placement.

Major 5. The authors should independently quantify the plasmid stock concentrations using a validated singleplex dPCR and an orthogonal method like fluorometry. Using these well-characterized standards, a full assay performance evaluation should be conducted, including accuracy, repeatability, intermediate precision, linearity, and the reportable range.

We would like to clarify that all plasmid stocks concentrations were independently quantified by fluorometry prior to use in the study. We regret that this information was omitted from the original manuscript and have now added this clarification to the Methods section. Each plasmid preparation was normalized to a targeted concentration of 1x1010 copies/ml based on the fluorometry measurement and plasmid template length using the formula: ([plasmid DNA ng/ml] * 6.022 x1023)/ (plasmid length in base pairs * 1 x109 ng/g * 650 g/mol) = copy number. During quadplex assay development, these plasmid stock concentrations were measured in singleplex assays.

We agree that assessment of accuracy, repeatability, intermediate precision, linearity, and reportable range is important for assay validation. However, the objective of this study was development and characterization of a quadplex linkage model rather than formal assay qualification. To characterize conditions supporting practical application of the model, we first evaluated the recovery of individual target concentrations in quadplex reactions across a broad concentration range (Fig. 2, Table 1). Target recoveries remained within the predefined 80-120% interval from approximately 39-5000 copies/µL for the intact plasmid template. Subsequent experiments demonstrated that this target-recovery range does not constitute a universal operating range for the linkage model because the model performance also depends on total droplet occupancy, template composition, fragmentation state, and primer-probe design. Accordingly, we view the experiments presented in this manuscript as a framework for defining model limitations and establishing the types of control studies that should be performed when adapting the approach to a specific application. We have revised the manuscript to emphasize that users should empirically evaluate model performance using representative materials and experimental conditions relevant to their intended use rather than applying a universal operating range derived from this study.

Minor 1. The description of rcAAV in the second paragraph of the Introduction is somewhat brief. The authors should briefly describe its origin, its impact on product safety, and the principles of conventional detection methods, such as cell-based amplification assays, to provide readers with better context.

We thank the reviewer for this comment. We recognize that our original wording may have caused confusion. The intent of this paragraph was to discuss analytical challenges associated with recombinant AAV (rAAV) vector heterogeneity, genome integrity, and production-derived DNA impurities and was not intended to refer to replication-competent AAV (rcAAV). The quadplex linkage assay described in this manuscript is intended to characterize nucleic acid integrity and target linkage, and is not designed to detect or quantify rcAAV. To improve clarity, we have revised the paragraph to more explicitly focus on rAAV product heterogeneity, genome integrity, and production-derived impurities. As rcAAV detection is outside the scope of the present work and is not an intended application of the method, we have not expanded the Introduction to include a discussion of rcAAV biology or conventional rcAAV detection assays.

Minor 2. Including the actual fragment lengths in base pairs in Table 2 would help readers better understand the discussion on how fragment size affects model accuracy.

We thank the reviewer for this suggestion. We agree that including the fragment lengths provides additional context and have revised Table 2 to include the expected fragment lengths (bp) associated with each digestion condition. Additionally, we have revised the Results and Discussion to clarify our experimental data most directly supports occupancy as the primary empirical factor affecting model performance. Fragment size and occupancy were not independently controlled in the present study, so we cannot conclude that fragment length itself is an independent driver of model accuracy.

Minor 3. The authors should provide additional details on fluorescence compensation, cluster definition, and threshold setting, and include representative two-dimensional cluster plots to demonstrate assay specificity.

We thank the reviewer for this suggestion. We agree that additional detail regarding threshold setting and cluster classification improves interpretability of the assay workflow. We have expanded the Methods section to clarify that amplitude-based positive/negative droplet thresholds for each channel were established following qualitative review of sample and no template control wells within the QX ONE software. To avoid introducing analyst bias, identical thresholds were applied across all wells within an experiment. No additional user-applied fluorescence compensation was performed. Following thresholding, droplets were automatically classified by the QX ONE software into the 16 possible four-channel binary cluster states (0000-1111). Two datafiles were finally exported from the QX ONE software (Visible Data and Cluster Data). Both export files were used as inputs for the quadplex model.

Candidate primer/probe sets were screened during assay development for adequate amplitude separation and minimal channel crosstalk. Representative 1D and 2D droplet plots have been included in the Supporting Information (S1-S6 Figs) to demonstrate assay specificity and cluster separation. Representative 2D ddPCR plots for AAV9-BIIB1 and AAV2-BIIB2 have been added as supplemental figures (S15-S20 Figs) for transparency.

Minor 4. Language and terminology require editing throughout the manuscript. For example, "lipid nano particles" should be "lipid nanoparticles," and the use of units like "µL" and the capitalization of "Bio-Rad" should be made consistent.

We thank the reviewer for these suggestions and have corrected these inconsistencies throughout the revised manuscript.

Minor 5. The serotypes of AAV-BIIB1 and AAV-BIIB2 are not mentioned anywhere. Different serotypes have different capsid stabilities and genome release properties, which could affect decapsidation efficiency and consequently the model's estimates. It would be helpful to specify this.

We thank the reviewer for this suggestion and have added the serotypes as clarification throughout the revised manuscript.

Minor 6. In the first paragraph of the Discussion, the authors state "While triplex ('3D') linkage analysis has been recently published, the statistical framework was not fully described [32]." The authors should also consider PMID: 39820513. Additionally, in the second paragraph of the Introduction, where the authors discuss rAAV vector quality attributes, the citation of PMID: 38307819 is recommended providing more comprehensive literature support for the background on rAAV vector characterization and quality control.

We would like to clarify that the reviewer’s suggested reference for 3D linkage analysis PMID: 39820513 was already included in the manuscript as reference 32. We reviewed PMID:38307819 and appreciate the recommendation. Since the study focuses on capsid engineering to enhance rAAV transduction rather than genome integrity, product heterogeneity, or analytical characterization we did not find it directly relevant to the Introduction and have not added it as a citation.

Minor 7. The authors note in the Methods that a linear mixed-effects model with replicate as a random effect was evaluated, but only Pearson's r and R² are reported in the main text. Since the mixed model was run, it would be good to present those results as well.

We have revised the Results section and Figure 7C to include the linear mixed-effects model results in addition to the linear regression. Consistent with the simple linear regression (solid line, Pearson r = 0.91, R² = 0.82), the mixed-effects model demonstrated a similar relationship between intact genome probability (p1111) and maximal protein expression (Emax) after accounting for replicate-level variation (dashed line, β = 5.98, p < 0.0001).

Response to Reviewer #2

Major 1. The authors empirically establish a concentration range (39- 5000 copies/μL) and a negative droplet threshold (≥5000" " empty droplets) required for accurate model performance. While these operational recommendations are practical, using an absolute count of 5000 empty droplets lacks statistical rigor because the total number of accepted droplets (N) varies between wells/reactions (typically around 20,000). Therefore, an absolute cutoff of 5000 empty droplets does not hold consistently across varying total droplet counts. For the fitting plot in Figure 5, I strongly suggest re-parameterizing the x-axis using the proportion of positive droplets (k/N), the proportion of negative droplets ((N-k)/N), or the overall occupancy parameter λ.

We agree that expressing the Figure 5 threshold solely as an absolute number of empty droplets is not generalizable across samples because the total number of accepted droplets varies across wells. To align with your suggestion, we re-parameterized the Figure 5 x-axes using normalized occupancy-based quantities: the empty-droplet fraction ((N-k)/N), the positive droplet fraction (k/N), and the occupancy parameter λ = -log((N-k)/N). The elbow analysis was performed on the empty-droplet fraction and the corresponding reference positions for positive-droplet fraction and λ were obtained by algebraic transformation of the same threshold. We have revised Fig 5 and the accompanying Results section to emphasize empty-droplet fraction and occupancy as the more generalizable performance metrics. We have reanalyzed the rAAV datasets with the identified ≥27.6% empty-droplet fraction threshold. This change affected the inclusion of one well and did not alter the reported conclusions. The Methods section and Fig 7 have been revised to reflect the updated data-filtering criterion.

Major 2. The presentation of probability estimates in Figures 3 and 4 is somewhat confusing. For any given sample, should the sum of all modeled probabilities across all species equal 1 (100%)? If the model output sums to greater or less than 1, are these values normalized back to 1? Please clarify how probability constraints and normalization are mathematically operationalized within the algorithm, particularly when impossible or negative fragment probabilities arise.

We thank the reviewer for highlighting this point. In Figures 3 and 4, the plotted values are not all intermediate model outputs; rather, they comprise the singlet species, the experimentally permitted linked intermediate species for a given probe order, and the derived intact species (p1111). Intact probability is calculated as the remainder after summing the linked non-intact species, i.e. p1111 = 1 - Σ p(linked non-intact species). Thus, when the model behaves well, the displayed probabilities for a given well are intended to sum to 1. We do not apply any post hoc renormalization to force the outputs back to 1. This is intentional: deviations from the [0,1] interval are treated as indicators of model instability under nonideal occupancy rather than being hidden by rescaling.

Operationally, component probabilities obtained by polynomial solving are constrained to real roots in [0,1]; impossible configurations return 0 or NA. Negative values therefore do not arise for the component fragment probabilities themselves, but this derived intact remainder can become negative if the summed non-intact probability mass exceeds 1, which we interpret as a failure mode of the estimator. We have added clarification around this point to the Results section.

Major 3. In Section III (p. 12) and the Discussion (p. 18), the authors hypothesize that smaller template fragments physically fit into droplets in greater numbers than larger fragments, leading to model inaccuracy. However, the current experimental design does not directly isolate fragment size (e.g., comparing long fragments of several kilobases against short fragments of a few hundred base pairs at identical molar concentrations). From the raw data, the higher-fragmentation samples simply contain significantly lower empty droplet counts. This suggests that the observed bias may be driven by high target occupancy (λ) rather than physical exclusion/size effects. The authors should clarify this distinction and consider whether λ or the positive droplet ratio (k/N) is the primary governing parameter.

We agree that the current study was not designed to isolate fragment size independently at matched molar occupancy and therefore does not directly demonstrate a fragment-size effect apart from occupancy. Our validation data most directly support droplet occupancy (λ) as the empirical parameter associated with model performance, with lower empty-droplet fractions and higher λ values accompanying the conditions in which unstable estimates emerged. We interpret fragment size as a plausible contributing explanation for those occupancy differences rather than as an independently proven driver in the present dataset. Therefore, we revised the discussion to clarify that model performance appears to depend on the combined effects of template fragmentation and droplet occupancy and note that these variables were not independently controlled in the present study. Additionally, Fig 5 plot axes were revised to occupancy metrics (as described in Major Comment 1).

Major 4. The Discussion should expand on how total target occupancy (λ) and negative droplet ratios govern model accuracy. The concentration dynamic range established for a single-target assay (39-5000 copies/μL) does not directly translate to multi-target quadplex assays, where the cumulative concentration of all fragments combined determines the total partition occupancy and false co-encapsulation rates.

We agree with the reviewer that the concentration range established from these validation experiments should not be interpreted as a universal single-target rule for quadplex assays. We have revised the Results and Discussion sections to emphasize droplet occupancy as the main empirical driver of model performance, while any direct fragment size effects remain hypothetical and not fully explored in this study. We would like to clarify that the lower concentration threshold of 39 copies/μL was not derived from a single-target assay, but rather, is based on the percent recoveries of single targets within quadplex reactions. Regardless of this minor point, we have revised the Results section to clarify this lower limit was empirically derived for the intact plasmid and quadplex panel evaluated and should not be interpreted as a universal boundary.

Minor 1. In the raw dPCR data, several assays exhibit low-level false-positive droplets in non-template control (NTC) wells. Are these false positives attributable to primer-dimer formation, probe crosstalk, or sub-optimal thresholding settings?

We thank the reviewer for this observation. Low-level positive events were observed in NTC wells for some assays and appeared to be quadplex-panel dependent. NTC background was more pronounced with the AAV2-BIIB2 quadplex primer/probe panel, particularly for the WPRE target which suggests an assay-specific source of background. Potential contributors include low-level non-specific amplification, probe-associated background, or inter-channel interference. We have therefore identified the panel- and target-dependent nature of the observation without assigning a definitive mechanism. During assay development, we screened multiple quadplex combinations and selected the configurations that provided the best overall performance. However, due to the limited size of the AAV genome, practical constraints on target placement can limit optimization of multiplex reactions. We acknowledge this as a limitation of the approach and have revised the manuscript to discuss this limitation and advise caution when implementing panel designs.

Minor 2. The starting stock concentrations for plasmid preparations are relatively low (∼0.04 ng/μL). Were specific precautions taken to prevent non-specific surface adsorption during serial dilutions (e.g., using low-binding plasticware or adding carrier nucleic acids such as 10 ng/μL yeast tRNA)?

We thank the reviewer for this comment and apologize for omitting this clarification from the Methods section. The Methods section has been revised to state that a final concentration of 10 µg/ml sheared salmon sperm DNA was added to each diluted plasmid working stock to act as a carrier DNA and reduce template loss due to adsorption during serial dilution. In addition, dilution buffers contained Pluronic F-68 (poloxamer 188), which was included to further reduce nonspecific adsorption of nucleic acids to plastic surfaces.

Minor 3. Although "ddPCR" is widely recognized, the manuscript references digital PCR technology more broadly. To maintain precise distinction between microfluidic/nanowell-based dPCR and droplet-based partitioning, consider using "droplet dPCR" or "dPCR" consistently throughout the text instead of mixing "ddPCR" and "dPCR".

We agree that inconsistent use of the terms "dPCR" and "ddPCR" may be confusing to readers. To improve clarity, we have revised the manuscript to use “droplet dPCR" as recommended, to consistently refer to the droplet-based platform experiments performed in this study.

Minor 4. Check typos in target parameter names throughout Section II and the manuscript text (e.g., pPuroR_AmpR vs. ppuroR_ampR).

We thank the reviewer for this suggestion and have corrected these inconsistencies throughout the revised manuscript.

Attachment Submitted filename: Response to Reviewers.docx

# Peer review: Decision Letter 1

**DOI:** 10.1371/journal.pone.0362653.r004

**Authors:** Chen Ling

28 Oct 2026

<p>A Poisson-multinomial model for quantifying gene therapy vector integrity using quadplex digital PCR

PONE-D-26-28635R1

Dear Dr. Tereshko,

We’re pleased to inform you that your manuscript has been judged scientifically suitable for publication and will be formally accepted for publication once it meets all outstanding technical requirements.

Within one week, you’ll receive an e-mail detailing the required amendments. When these have been addressed, you’ll receive a formal acceptance letter and your manuscript will be scheduled for publication.

An invoice will be generated when your article is formally accepted. Please note, if your institution has a publishing partnership with PLOS and your article meets the relevant criteria, all or part of your publication costs will be covered. Please make sure your user information is up-to-date by logging into Editorial Manager at Editorial Manager® and clicking the ‘Update My Information' link at the top of the page. For questions related to billing, please contact billing support.

If your institution or institutions have a press office, please notify them about your upcoming paper to help maximize its impact. If they’ll be preparing press materials, please inform our press team as soon as possible -- no later than 48 hours after receiving the formal acceptance. Your manuscript will remain under strict press embargo until 2 pm Eastern Time on the date of publication. For more information, please contact onepress@plos.org.

Kind regards,

Chen Ling, Ph.D.

Academic Editor

PLOS One

**Comments to the Author**

Reviewer #1: All comments have been addressed

Reviewer #2: All comments have been addressed

**********

2. Is the manuscript technically sound, and do the data support the conclusions?

Reviewer #1: Yes

Reviewer #2: Yes

**********

3. Has the statistical analysis been performed appropriately and rigorously?

Reviewer #1: Yes

Reviewer #2: Yes

**********

4. Have the authors made all data underlying the findings in their manuscript fully available?

The PLOS Data policy

Reviewer #1: Yes

Reviewer #2: Yes

**********

5. Is the manuscript presented in an intelligible fashion and written in standard English?

Reviewer #1: Yes

Reviewer #2: Yes

**********

Reviewer #1: The authors' responses are of high quality and include several key additional experiments. I consider the manuscript acceptable for publication. The following points may be considered as optional additions:

In the second paragraph of the Introduction, given that rcAAV is an important consideration for rAAV product safety and that its detection methods provide a complementary background to the analytical characterization challenges discussed in this paper, the authors may add a brief introduction to rcAAV and cite several relevant articles on rcAAV, including but not limited to PMID 41020265, PMID: 40777727, etc.

Reviewer #2: (No Response)

**********

what does this mean?). If published, this will include your full peer review and any attached files.

If you choose “no”, your identity will remain anonymous but your review may still be made public.

**Do you want your identity to be public for this peer review?**  For information about this choice, including consent withdrawal, please see our Privacy Policy

Reviewer #1: **Yes:**  Chen Ling

Reviewer #2: No

**********

# Editor report: Acceptance letter

**DOI:** 10.1371/journal.pone.0362653.r005

**Authors:** Chen Ling

PONE-D-26-28635R1

PLOS One

Dear Dr. Tereshko,

I'm pleased to inform you that your manuscript has been deemed suitable for publication in PLOS One. Congratulations! Your manuscript is now being handed over to our production team.

At this stage, our production department will prepare your paper for publication. This includes ensuring the following:

* All references, tables, and figures are properly cited

* All relevant supporting information is included in the manuscript submission,

* There are no issues that prevent the paper from being properly typeset

You will receive further instructions from the production team, including instructions on how to review your proof when it is ready. Please keep in mind that we are working through a large volume of accepted articles, so please give us a few days to review your paper and let you know the next and final steps.

Lastly, if your institution or institutions have a press office, please let them know about your upcoming paper now to help maximize its impact. If they'll be preparing press materials, please inform our press team within the next 48 hours. Your manuscript will remain under strict press embargo until 2 pm Eastern Time on the date of publication. For more information, please contact onepress@plos.org.

You will receive an invoice from PLOS for your publication fee after your manuscript has reached the completed accept phase. If you receive an email requesting payment before acceptance or for any other service, this may be a phishing scheme. Learn how to identify phishing emails and protect your accounts at https://explore.plos.org/phishing.

If we can help with anything else, please email us at customercare@plos.org.

Thank you for submitting your work to PLOS One and supporting open access.

Kind regards,

PLOS One Editorial Office Staff

on behalf of

Dr. Chen Ling

Academic Editor

PLOS One
